# TELMA-PSYK - A Digital Homebased Research and Treatment Application in the Mental Health Service

**DOI:** 10.1192/j.eurpsy.2025.1430

**Published:** 2025-08-26

**Authors:** N. M. B. Johansen, S. F. Austin, A. A. Shaker, S. M. Arnfred

**Affiliations:** 1 Psychiatric Research Unit, Region Zealand Psychiatry, Slagelse, Denmark

## Abstract

**Introduction:**

The gap between staff resources and population needs for psychiatric treatment and support, as well as the geographical gap between services delivered in rural areas and in cities is a major challenge for the Danish society as well as globally.

The current project will investigate video consultations (VC) in the context of developing a full-scale home-based mental health service for users with severe mental illness (SMI) necessitating multidisciplinary and municipal support. Digital patient reported outcome (PRO) monitoring can strengthen shared decision making in consultations, and is frequently applied for users with SMI, but it is not regularly combined with VC.

TELMA (Telemedical Monitoring Application) is a national IT system that makes it possible to monitor a patients’ health status in their own home. TELMA includes several questionnaires and the option to connect telemedicine devices that can send vital data such as sleep quality or heart rate measurements directly to the app using Bluetooth. A number of affiliated, cross-sectoral teams who monitors the patient’s health status can view the patient’s responses and data. By mapping the needs and preferences of patients and staff along with an initial trial of the application of TELMA-PSYK for home-based treatment in psychiatry, this study will pave the way for the development of fully digital, person-centered, sector integrating home-based treatment.

**Objectives:**

This study aims to explore barriers and opportunities to home-based treatment for users with severe mental illness as seen by stakeholders, staff, management and users in mental health services and municipalities.

**Methods:**

TELMA-PSYK will be examined in a small-scale feasibility study.

Twenty users with SMI and their regional and municipal staff will be invited to participate in the study. A qualitative study based on phenomenological enquiry will capture the respondents’ experiences of VC and their concerns for and opportunities of full-scale home-based mental health service with PRO recording.

Qualitative data will be interviews examining the users’ experience of three months’ home-based service provision, and the corresponding staffs’ experience of the user’s treatment course and the intervention. Qualitative data will be analyzed with reflexive thematic analysis.

**Results:**

It is expected results from the feasibility study will be ready by March 2025.

**Conclusions:**

It is expected conclusions from the feasibility study will be ready by March 2025.

**Disclosure of Interest:**

None Declared

